# Parent and Peer Emotion Responsivity Styles: An Extension of Gottman’s Emotion Socialization Parenting Typologies

**DOI:** 10.3390/children8050319

**Published:** 2021-04-22

**Authors:** Jens E. Jespersen, Nathan R. Hardy, Amanda Sheffield Morris

**Affiliations:** Department of Human Development and Family Science, Oklahoma State University, Stillwater, OK 74078, USA; nathan.hardy@okstate.edu (N.R.H.); amanda.morris@okstate.edu (A.S.M.)

**Keywords:** parents, peers, emotion, emotion socialization, emotion responsivity styles

## Abstract

This theoretical paper introduces six emotion socialization typologies that can be used for designating emotion responsivity styles of parents and peers of children in middle childhood, referred to as Parent and Peer Emotion Responsivity Styles (PPERS). This typology draws on theoretical foundations of meta-emotion and emotion socialization. These typologies are compliment with and extend Gottman’s emotion-based parenting styles, as they are organized generally by whether the response is more positive or more negative and whether the response is more emotionally constructive or destructive, but extend the four styles to include whether the parent or peer targets the emotion directly when responding to a child’s emotions, or whether they target the emotion-related behavior. On the positive end, there is the Emotion Constructive style, which targets the child’s emotions directly. The other two positive styles include Emotion Responsive and Emotion Acceptive, which target the child’s emotional behaviors with higher or lower levels of activity. On the negative side, there is the Emotion Destructive style which is employed to target the emotion itself, while the Emotion Punitive and Emotion Dismissive styles target the child’s emotion-related behavior with varying levels of activity. Implications for the development and study of these theoretical typologies are discussed.

## 1. Introduction

Over the past 20 years, researchers have advanced our understanding of emotion-related behavior and emotion socialization, contributing to a recognition of emotion socialization as a key factor for optimal development throughout childhood and across the lifespan [1,2]. Along with increased recognition in the popular press and academic literature, scholars have developed a number of ground-breaking theories [3,4,5,6] and models [7,8] that have guided research efforts and understanding of when, how, and why children’s emotion related behavior is developed and socialized, particularly in the context of emotion socialization primarily by parents [7], but also by peers [2]. Through these advancements in theory and research it has become clear that parents and peers play a notable role in the socialization of emotion in children. Moreover, it has become increasingly recognized that parents respond or react to their children’s displays of emotion through various pathways and contexts [7,8] and in ways that can be categorized into general typologies [5]. In this manuscript, we carry out a scoping review of relevant literature and propose emotion socialization typologies, Parent and Peer Emotion Responsivity Styles (PPERS). This typology focuses largely on the manner in which parents respond to the emotions of their young children, and how children respond to the emotions of their peers. Such a typological approach can be useful in discussing and organizing future socio-emotional research and for practitioners who work with families and children on emotion-related difficulties.

The imperative for further studying the phenomenon of parent and peer emotion responsivity is grounded in the health and development of the child. Throughout the early stages of childhood, children begin to develop an awareness of the feelings that they experience, as well as how these feelings are presented and responded to by others—especially their primary caregivers, and eventually their peers [8]. As such, children possess an increased vulnerability to potentially damaging emotion responsivity styles that can shape their perception of emotions and guide their social and emotional development in ways that can be maladaptive and potentially contribute to psychopathological problems [9,10,11,12]. With parents being their child’s first teachers and models, and peers quickly becoming a second, it is important for children to be taught and responded to in ways that will nurture their emotional growth and cultivate their understanding of their feelings and the feelings of others [13]. 

**Figure 1:**
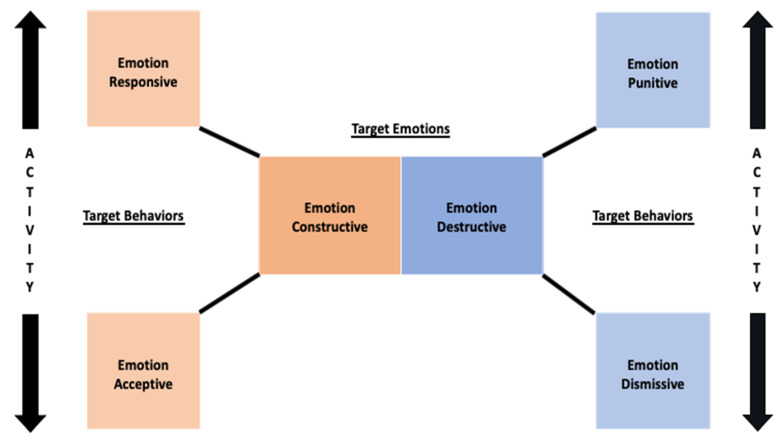
Parent and Peer Emotion Responsivity Styles. The central responsivity styles of Emotion Constructive (positive) and Emotion Destructive (negative) are employed to target emotions directly, while the outer styles target emotion-related behavior with varying levels of activity, with Emotion Responsive (positive) and Emotion Punitive (negative) featuring higher activity, and Emotion Acceptive (positive) and Emotion Dismissive (negative) utilizing less parental/peer activity.

This theoretical paper introduces six emotion socialization typologies that can be used for designating emotion responsivity styles of parents and peers of children in early and middle childhood (ages 4–9). This theory is developed for children in this age range as children at this stage begin to experience enhanced emotional-communication ability, and increasingly rely on internal cognitive coping strategies rather than relying primarily on external regulation [8]. This theory draws on principles of meta-emotion, which is “an organized and structured set of emotions and cognitions about one’s own emotions and the emotions of others” [4] (p. 7), with an understanding that parents and peers tend to have emotions about children’s emotions and emotional expressions based on their own individual values, perceptions, or feelings [3]. Although Baumrind presented what we know to be the traditional parenting styles (i.e., authoritative, authoritarian, permissive, uninvolved; [14]), Gottman is credited for categorizing parenting styles based on emotion socialization typologies (i.e., emotion coach, dismissing, disapproving, laissez-faire; [5]). The PPERS typologies presented in this paper both compliment and extend Gottman’s traditional parenting styles of emotion socialization, in that they are organized generally by whether the response is more positive or more negative and whether the response is more emotionally constructive or destructive, and extends the typologies to include whether the parent or peer target the emotion directly when responding to a child’s emotions, or whether they target the emotion-related behavior. These differentiations, as well as the addition of a theoretical typology that can be used for categorizing peer emotion socialization styles go beyond Gottman’s original theoretical presentation while also contributing a model not previously existing within the child peer literature. On the positive end, there is the Emotion Constructive style, which targets the child’s emotions directly. The other two positive styles include Emotion Responsive and Emotion Acceptive, which target the child’s emotional behaviors with higher or lower levels of activity. On the negative side, there is the Emotion Destructive style which is employed to target the emotion directly, while the Emotion Punitive and Emotion Dismissive styles target the child’s emotion-related behavior with varying levels of activity (see Figure 1). This extension to Gottman’s parenting typologies is warranted as these additional typologies create a more dynamic perspective that includes the differentiation between emotion- and behavior-based responses, while additionally contributing to the potential examination of the role of peer-based emotion responsivity in early childhood, a research area that is currently less understood [2,7]. 

This theoretical approach can enhance the field’s understanding and organization of how children’s emotions are responded to, and the social and emotional consequences of different responsivity styles. Additionally, the use of this typology in context of child peers introduces a tool for categorizing peer emotion socialization styles not previously existing within the child peer literature. Furthermore, this typology may contribute to additional speculation into mechanisms or pathways whereby children are emotionally socialized by their parents, as well as how they internalize emotion responsivity styles and use them in emotion-based child–peer interactions. Moreover, methods of measurement as well as implications for the development and study of these theoretical typologies can affect future research.

### 1.1. Emotion Socialization

To begin laying the foundation for these theoretical typologies, we first identify the core construct of emotion socialization. Emotion socialization is broadly defined as social behavior that influences how a child learns about and comes to exhibit emotion-related behavior, including emotional experience, expression, and regulation [7]. The parents, peers, and child each play a notable role in the context of emotion socialization and subsequent emotion-related development and behavior. Considering Sameroff’s Transactional Model [15], emotion socialization can be considered a bidirectional process that is guided by both the socializer and recipient’s emotional, behavioral, and genetic characteristics. Furthermore, by adding the perspectives of Bronfenbrenner’s process-person-context-time (PPCT) model [16], emotion socialization is additionally influenced by the context of the interaction and where it fits within the timeline of development and appropriateness within society [16,17]. For young children, the most salient and influential emotion socializers tend to be primary caregivers, with peers, teachers, and other adults increasing in influence as the child gets older [18]. Moreover, emotion socialization begins early in infancy, where early interactions with primary caregivers establish a foundation from which children gradually develop emotional autonomy and are eventually capable of regulating their own emotions [7,8]. 

### 1.2. The Role of Parents in Emotion Socialization

Parents are a child’s first teacher, example, and socializer. Consequently, children’s first exposure to a range of emotional experiences most often comes from their primary caregivers [19]. Eisenberg and colleagues [7] posited that children are emotionally socialized by their parents through three primary pathways, including parents own expressions of emotion, parents’ reactions to the emotions of their child, and through parents’ discussion of emotion with their child. Considering the first pathway of parents’ emotional expressiveness, it can be understood that parents shape the emotional schemas of their children via their own emotional expression. Through these schemas, children attribute context and meaning to emotional expression, as well as develop an idea of what form of emotional expression would be considered “appropriate” in a given situation. The second pathway concerns emotion-based discussions between parent and child, which can aid children in understanding how emotion fits into social contexts [20]. The third pathway is focused on parents’ reactions or responses to the emotions of their children. These reactions serve to emotionally socialize the child in their own emotional understanding, expression, and regulation. Parents’ reactions have been found to either support positive emotional expression and regulation through coaching and scaffolding, or to diminish emotional expressiveness through minimization or punishment of an emotion or emotion-related behavior [3,21]. 

Beyond Eisenberg’s model of emotion socialization, it has been well documented that children begin learning about emotions by observing the emotions of their parents [7,8]. As such, parents offer an abundance of opportunities for their child to observe a wide range of emotional expressions that can vary in frequency and intensity. Beyond simple observation, children also begin to look to their parents for guidance in how to express their feelings, as well as ways to interpret emotional and environmental cues from others [22]. This social referencing can be accomplished through discussion with parents, or by observing how parents tend to respond to both the child’s emotions and the emotions of others [19,23]. As children develop the ability to converse in early childhood, parents begin to be able to discuss emotions or emotion-related situations with their child, which can contribute to enhanced emotion knowledge and increased emotion socialization [24]. In addition, as children progress from early childhood into middle childhood they are more capable of developing cognitive strategies that allow them to express, internalize, and regulate their emotional experiences and behavior with increasing independence, though parents often continue to act as essential co-regulators by providing consistent emotional guidance and support [8,25]. As such, the dynamic process of emotion socialization progresses through parent–child interactions, and eventually through interactions with peers [26].

### 1.3. The Role of Peers in Emotion Socialization

While the model of emotion socialization presented by Eisenberg and colleagues tends to focus on parent–child relationships, they acknowledge that the broader socialization literature provides evidence for other influential socialization figures outside of the home environment, stating that “There are many socializing forces besides parents, including siblings, peers, and teachers” [7] (p. 267).

As children reach school age, they begin exploring unfamiliar environments that offer new relationships, challenges, and responsibilities. In these novel environments, children are in frequent contact with new emotion socializers, including teachers, other adults, and their peers [27]. During the preschool years and throughout middle childhood, increased school attendance contributes to children spending more time away from home, resulting in less frequent interaction with their parents. Increased emotion socialization is likely to take place within school settings as children spend upwards of 7 h per day, for the greater part of the year, in the classroom [28]. In school settings, children frequently experience and observe a wide range of emotions throughout the day, including anxiety regarding new situations and expectations, sadness stemming from disappointment or difficult social interactions, frustration from potentially difficult subject matter, and happiness from satisfying social experiences and achievement [2,29]. The timing of school entry is also of note, as children at this age have entered a developmental period where they tend to possess a heightened awareness for and interest in the contextual appropriateness of emotions and emotion-related behaviors [30,31]. As such, children begin to look beyond the parent–child relationship for emotional guidance and strategies for regulation, in addition to the resources and examples that they have at home [27]. Due to the finding that children tend to place increased value on their peer interactions as they get older, as well as how they have been found to match their emotional behaviors to those of their peers, peers are believed to play a notable role in the emotion socialization of children through peer influence [32,33], although this has received much less attention in the literature than parent emotion socialization. While these findings offer a stable foundation to build upon, additional research and theory development are needed to further investigate the role of peers in emotion socialization, particularly in early and middle childhood [29,32]. 

## 2. Key Concepts and Propositions

The Parental Emotion Responsivity Styles draw heavily from Gottman’s Four Parenting Styles of Emotion [5]. We posit that as a whole, the PPERS are an extension or reframing of Gottman’s work. Of the six emotion responsivity types discussed in the PPERS, three of them were originally presented by Gottman (emotion coach, dismissing, and disapproving). However, as it will be discussed moving forward, these emotion-based responsivity styles are categorized differently and observed according to positivity and negativity of response, activity level, and whether responses are emotion- or behavior-based. To broadly set the stage, we conceptualize emotion responsivity styles as being either more positive or more negative. The six emotion responsivity styles have been broken down into two general groups, with one group responding to emotions in a manner that is generally warm, and the second group representing responsivity styles that are generally negative, or cold. Within each of these two general groups exist three primary responsivity styles that are categorized by whether the responsivity style directly targets emotions themselves, or whether they are in response to emotion-related behavior. Those responsivity styles related to emotional behavior are differentiated by activity, or, whether emotion socializers make an active effort or tend to be more “hands-off” (see Figure 1). We posit that children who are regularly exposed to these emotion responsivity methods will eventually incorporate similar emotional responsivity styles as they interact with and respond to the emotions of their most prominent emotion socializers, including their parents and their peers.

### 2.1. Positive Responsivity Styles

#### 2.1.1. Emotion Constructive

This responsivity style has largely been adopted from Gottman’s Emotion Coach parenting style [5], as individuals who utilize this style tend to address emotions themselves when interacting with a child who is having an emotional moment, by doing things such as listening empathetically, validating the child’s feelings, and helping the child label their emotion in order to aid them in understanding why they might be feeling the way they do. This responsivity style provides emotion-based teaching, modeling, and scaffolding to build emotion regulation skills. For example, themes of this responsivity type have been studied by examining how emotion-based coaching and scaffolding contribute to child emotion regulation. Using functional near-infrared spectroscopy (fNIRS), Grabell and colleagues [34] found these practices to be associated with enhanced activation of the child’s lateral prefrontal cortex, indicative of improved emotion regulation. Additionally, Leventon and colleagues [35] found that discussing emotional experiences can contribute to lower neural reactivity to emotions in children. Despite limited findings in the context of child peer socialization, this emotion responsivity style has been found to be more often employed by girls than boys, as girls tend to emphasize emotional intimacy, disclosure, and validation in their responses to peer emotions [36]. In sum, parents and peers who use this approach seek to help the child become positive emotion regulators, with emotion being considered appropriate and adaptive. 

#### 2.1.2. Emotion Responsive

Beginning with the more “active” of the positive behavior-targeted styles, we have the Emotion Responsive style. This style was drawn out of the behavior-based components of Gottman’s Emotion Coaching parenting style [5] to create a warm and positive style that targets behavior, rather than both behavior and emotion. Instead of directly addressing the emotion with the children, parents who utilize the Emotion Responsive style actively respond to their child’s displays of negative emotion with warmth, and try to soothe, distract, or cheer them up, but do so without labeling or discussing the emotion being experienced. This responsivity style has been found to be associated with enhanced effortful control and decreased externalizing problems in children [11,12]. Additionally, positive behavior-based emotion responsivity has been associated with better behavior-based emotion regulation skills over time [37]. In the context of child peers, this responsivity style has been found to be utilized more often by boys, as boys tend to respond to emotional behavior with deeds and actions rather than more intimate emotional discussions [38]. While this style is warm and positive, individuals who use this style tend to use an external approach, targeting the outcomes of emotionality rather than getting directly to the core of the emotions the child is feeling and why.

#### 2.1.3. Emotion Acceptive

This responsivity style is the less active positive behavior-based style, and was derived from Gottman’s Laissez-Faire parenting style [5]. Similarly, parents who are Emotion Acceptive do not judge or condemn their children for displaying behavior associated with negative emotions, but embrace and accept whichever emotions their child might be feeling. With this responsivity style, Emotion Acceptive parents allow the child to work through their feelings with limited guidance or aid, using an accepting, and more of a “hands off” or distant approach. Interestingly, evidence of this responsivity style has been inconsistent, with acceptance of behavior-based emotionality contributing to positive child outcomes (i.e., fewer internalizing problems) with lower effect than outright non-supportive responsivity, which has contributed to child internalizing problems more convincingly [10,11]. This indicates that detached approval may be less effective than detached disapproval in terms of emotional behavior modification. Moreover, in the context of peers, it has been found that child peers tend to respond to their friends’ emotional displays with less intensity as they age [39] suggesting that child peers may become less “active” in their emotional responses in their teenage years compared to middle childhood. 

### 2.2. Negative Responsivity Styles 

#### 2.2.1. Emotion Destructive

This concept adopts the emotion-focused elements from Gottman’s parenting styles of Dismissive and Disapproving [5] in order to generate one single negative responsivity style that is purely emotion-based, rather than the emotion- and behavior-based blend that is present in those two styles as presented in Gottman’s work. Individuals who utilize the Emotion Destructive responsivity style address the emotion itself, but attribute “undesirable” emotions to the child’s personal attributes of being irrational or weak. In this case negative emotions are considered inappropriate or maladaptive, and parents or children who utilize this approach seek to extinguish negative emotional expression. Outcomes associated with this responsivity style have been documented in multiple studies, where researchers used error-related negativity (ERN), a neural response to mistakes primarily measured using electroencephalography (EEG), to examine how harsh responsivity affected emotion-related neural function and structure [40]. In one such study it was found that children who received harsh responses to negative emotion possessed greater risk for adverse psychopathological outcomes, as indicated by increased levels of anxiety and negative affect [34]. In sum, parents and peers who use this approach seek to teach children that negative emotions are inherently wrong, with such emotions being considered inappropriate and maladaptive.

#### 2.2.2. Emotion Punitive

Moving out to the negative behavior-based responsivity styles, we have the more active of the two styles, Emotion Punitive. This parenting style is an adaptation of Gottman’s Disapproving parenting style [5]. Parents who employ this responsivity style actively respond to their child’s emotional behavior with punishment or criticism. These parents’ behavior regarding emotional displays might suggest that they believe that emotional behavior reflects poor character traits or a lack of personal control, and that the display of emotion, not necessarily the emotion itself, should be controlled or extinguished. Examples of this responsivity style have been examined by measuring emotion socialization practices of mothers of young children, where it was found that children of mothers who neglected or punished them for emotion-based behavior possessed elevated internalizing symptoms across the span of a year. Further, these findings were particularly prominent in children who already possessed high levels of internalizing problems [9]. Additionally, a study that used functional magnetic resonance imagining (fMRI) found that children of parents who tend to respond to emotions punitively were more reactive to emotional stimuli, as indicated by increased task-related amygdala activation [41]. In the context of child peers, it has been documented that child peers can be more likely to respond to a child’s negative emotional displays with more highly emotional negative responses than parents [39]. Taken together, these findings indicate that punitive responses to emotion-based child behavior can contribute to both enhanced psychopathological risk and maladaptive emotional functioning. 

#### 2.2.3. Emotion Dismissive

The final responsivity type is the more “hands off” negative behavior-based responsivity type, termed Emotion Dismissive. This responsivity type was developed using the external aspects of Gottman’s Emotion Dismissive parenting style [5]. Rather than making an active effort to squelch emotion-related behavior, individuals who employ this responsivity style tend to ignore, deny, or trivialize the child’s emotional behavior display in hopes that doing so will make the behavior go away on its own, or that the child will eventually “grow out of it” with the passing of time. This emotion responsivity style has been observed in studies of child emotion regulation, where it was found that children of parents who were unsupportive of their child’s emotional expressions possessed lower effortful control, increased externalizing problems, and poorer emotion regulation skills compared to children of parents who responded to their emotions supportively [11,12]. In a study of peer emotion socialization, it was found that children whose peers responded to their negative emotional displays with neglect were more likely to exhibit elevated levels of internalizing and externalizing problems [38], suggesting that negative peer responsivity may contribute to the development of psychopathology across childhood and into adolescence.

While each of these emotion responsivity styles are unique, we acknowledge that they are not mutually exclusive. It is possible or even likely that individuals may employ aspects of multiple styles in one response. For example, a parent could respond to an angry child by saying something like, “I can see that you are feeling angry, but it is not okay to hit your brother”. This example would suggest that this parent is employing both the Emotion Constructive and Emotion Responsive styles simultaneously. In this example, however, such an instance would be classified as Emotion Constructive, as the emotion itself was acknowledged. While parents and peers may use multiple responsivity styles in their responses, we posit that individuals will ultimately favor one style over another. 

There are a number of key propositions that we are making in presenting the use of this theoretical typology; the first being that parents’ and peers’ emotion responsivity styles are generally categorizable by extending Gottman’s four parenting styles. Gottman’s parenting styles of emotion have set a strong foundation for greater exploration into how parents respond to the emotions of their children. That being said, this foundation has great potential for further extension, and has not been applied to peers. As previously outlined, PPERS extends Gottman’s four parenting styles into six parent and peer responsivity styles, primarily by breaking them out into positive or negative emotion-targeted and emotion-behavior-targeted groups. This allows for more variation between Gottman’s four parenting styles that were in some ways unequally balanced. First, for example, the style of Emotion Coach is very broad, encompassing all positive elements of parents who are emotion-minded and actively involved, leaving little room for parents who are actively involved, but not necessarily emotion-minded. Dismissing and Disapproving styles appear to possess substantial overlap, and could logically be broken into additional categories based on activity level and whether the parents are emotion- or behavior-minded. Finally, the Laissez-Faire parenting style encompasses responses that did not fit into the three primary styles. We felt that this fourth style had greater potential and could be broken down by positive and negative attribution of emotion. By extending Gottman’s parenting styles we are proposing that more can be addressed in terms of parenting and peer responsivity styles, and that these responsivity styles have the potential to become more generally applicable, and potentially observable. 

The next key proposition that extends the applicability of Gottman’s theory is the key role that respondent activity level plays in distinguishing between behavior-based responsivity styles. Findings related to emotion responsivity or regulation style have illustrated that more “active” and attention-based strategies employed by parents are linked to enhanced desired outcomes, while passive strategies have been associated with lesser outcomes, as well as the development of externalizing behaviors [42,43]. The fact that there can be differences in activity or effort level with which parents or peers respond to a child’s emotion is clearly present, and appears to make a difference in children’s lives. This reality makes the proposed inclusion of activity level in developing responsivity styles an important factor to consider in our attempt to enhance the utility of Gottman’s parenting styles of emotion.

Finally, we propose that these responsivity types are best fit for categorizing parents and peers of children at a particular age/stage, while observing specific emotions. We posit that this theory would be most applicable with children in late early childhood and middle childhood, considering readily observable emotions such as anger or sadness [23]. As toddlers enter the phases of early and middle childhood, they experience development in executive function, inhibitory control, and language skills, making it possible for emotion socialization to be more problem solving and coping oriented [25]. During early and middle childhood, children still rely on their parents for aid in regulating their emotions, but they are beginning to understand that emotion is something they can start figuring out on their own [8]. This makes this time period the ideal window for observing how parents begin to verbally interact with their child and utilize whichever responsivity type they are more inclined to employ. Similarly, this is an ideal stage for examining these relationships at the peer level. Moreover, research has shown that children in early and middle childhood often rely more on cognitive emotion coping strategies rather than relying on physical comfort strategies that are more prevalent in infancy and toddlerhood [44], making the communication-based responsivity styles that we are presenting all the more prevalent. This may limit generalizability across age, but it should enhance the strength of this theory for children in early and middle childhood. 

## 3. Guiding Theoretical Frameworks

From a review of the existing parent and peer emotion socialization literature, the first author adapted and reorganized Gottman’s parenting styles to include additional styles, as well as application of this theoretical model to include both parents and peers. Moreover, this theoretical typology is influenced by aspects of a number of different theoretical frameworks, namely Reinforcement Theory, Social Learning Theory, Eisenberg et al.’s model of emotion socialization, Morris’ Tripartite Model of Emotion Regulation, and Meta-Emotion Theory. 

Reinforcement Theory [45] has long been regarded as one of the foundational psychological theories and has been applied to the study of emotion in a number of ways. For example, studies designed to evaluate the reinforcement of emotion suggest that emotions can be conceptualized as states that are produced and reinforced by various stimuli [46,47]. Reinforcement Theory would suggest that emotion and emotion related behavior can be developed or changed through reinforcement or punishment. A key theme of the PPERS is that parents and peers may be nurturing a child’s emotional growth through reinforcement, or they might be extinguishing emotion-related behavior through punishment or dismissal. This reinforcement or punishment based on emotional behavior could therefore have a generational effect as emotion responsivity styles are passed from parent to child, and even child to peer. 

The major themes of Social Learning Theory [48] are critical to the development of the PPERS theory because, as previously discussed, children have a propensity to learn how to feel about their emotions and the emotions of others by observing how their primary caregivers respond to emotions. While the majority of past research linking parent and child emotionality was conducted using biological family members, more recent findings have been successfully replicated using behavior-genetic designs, supporting the contribution of social learning to these parent–child emotionality links [19,49]. Similar to Reinforcement Theory, social learning pathways could potentially result in an intergenerational transmission of emotion responsivity. 

The next foundational framework is Eisenberg and colleagues’ model of parental socialization of emotion [7]. This theoretical model posits that the emotion socialization behaviors of parents, most importantly their expressions of emotion and reactions to emotion, have a direct influence on both their child’s level of emotional arousal as well as how their child learns about emotions. Additionally, this model ascribes parenting style as a major contributor to parents’ emotion socialization behaviors [7]. Of the three major pathways of emotion socialization outlined in this model, the existence of PPERS relies heavily on the third pathway—that children learn about appropriate emotional appraisal and expression by observing how others (parents, peers, etc.) respond to the emotions and emotion-related behavior of the child.

The fourth contributing framework is the Tripartite Model of Emotion Regulation [8], which posits that children’s strategies for emotion regulation are developed as they observe their parents’ emotion regulation practices, receive emotional guidance and coaching from their parents, and experience the emotional climate within their family. Considering this model, studies have shown that children of parents who coach them through their emotional experiences are more likely to possess enhanced emotion regulation skills [4]. Conversely, it has been found that children of parents who respond to their emotions negatively or punitively tend to have heightened emotional arousal [50]. This model can also be applied to peer relationships, as children experience the emotional climate of their relationship as well as observe the emotionality of others. 

Finally, Meta-Emotion Theory [3]. This theory is centered on emotions about emotions, or, how we feel about our feelings. This is a core component of PPERS, as this theory is built upon how parents and peers feel about the feelings of the child, and how they choose to respond to them, specifically. Foundational to PPERS is the acknowledgement that parents have feelings about their child’s emotions and emotion-related behavior, which can potentially lead to a parental response that may influence the child’s own feelings about their emotions or the emotions of others, which could then influence later behavior and emotion socialization of peers. Meta-Emotion Theory is valuable for recognizing how parents’ emotions about their child’s emotions can contribute to a chain reaction of parent behavior, emotional appraisal by the child, modified emotion-related behavior by the child, and the eventual emotion socialization of peers.

## 4. Theoretical Assumptions

In order for this theory to function and take root, there are a number of key assumptions that must be in place. First, it must be assumed that emotional expression is something that children do. Emotion is considered to be a universal phenomenon among human beings. From the earliest stages of life children are learning about their surroundings, their relationships, and themselves through the use of their emotions [51]. How these emotions are perceived by the individual child, as well as received by others (particularly primary caregivers and eventually peers), lays the foundation for social and emotional development that has been shown to develop rapidly over the early stages of one’s life, and contribute to behavioral patterns that are traceable across the lifespan [52]. With emotion development being such a focal point in the lives of young children, one of the major tasks in early and middle childhood involves learning about their various emotions, strategies for regulating them, and their use or effectiveness in a given situation [44]. 

Second, for these categorizable responsivity styles to exist it must be assumed that parents do, in fact, respond or react to their children’s emotional expressions and behavior, and that these interactions are observable. While many parents may respond or react to their child’s emotions differently than others, we present this theoretical typology under the assumption that parental emotion responsivity is universal behavior among parents of all cultures [53]. Not only does this assumption appear to exist, but it has also been associated with a number of implications, especially when considering how this interaction affects the child. A notable body of literature on parents’ responses or reactions to the emotions of their children suggests that these interactions play a very important role in the child’s understanding of emotion, as well as their socioemotional functioning [3,8,54] physiological arousal [3,21], and whether they will be passive or active when distressed [43,55]. 

Similarly, this theory assumes that peers respond or react to the emotional expressions and behavior of other children. Common peer socialization influences can take place within a school, within a classroom, within frequent interaction partners, and within friendships [15]. For the purpose of this theory, we focus on the more proximal peer socialization partners, including frequent interaction partners and friends. While less is known regarding the specific psychological pathways by which emotion socialization among peers tends to occur, the field has recently begun to investigate various mechanisms of socialization, including cognitive mechanisms via social comparison and self-evaluation; behavioral mechanisms through peer discussion, observational learning, and reinforcement; and socio-emotional mechanisms through establishing relationships. These mechanisms go beyond mere emotion contagion, as children actively seek these relationships which can guide and direct their emotion-related behavior [15]. For example, Cui and colleagues [50] found that emotionally supportive socialization practices by peers influences child affect, internalizing problems, and prosocial behavior over time. In addition to children socializing and being socialized by their peers, PPERS also assumes that children adapt similar emotional responsivity styles modeled by their parents, and that those emotion-based ideals and behaviors may even be passed from parents to peers, mediated by the child of the parent. As such, peers should not be viewed as independent generators of emotion responsivity. Rather, they may be socialized by their primary caregivers, and in turn socialize their peers based on modeling and reinforcement [7,56]. Furthermore, Criss and colleagues [23] suggest that both parents and peers play distinct roles in emotion socialization as children age, as they found peer interaction to be associated with variations in anger and sadness regulation in teenage girls, and parental emotional support and coaching playing a significant role in emotion regulation behavior. Therefore, the primary pathway whereby PPERS are developed move from parent to child, with the secondary pathway occurring between child and peers, keeping in mind that these pathways may change in salience as the child ages [57].

Finally, it must be assumed that parents and peers respond or react to other’s emotional expressions according to their own individual values, perceptions, or feelings about emotions. Theoretically, this assumption leans heavily on Gottman’s Meta-Emotion Philosophy [4], or, that individuals tend to possess emotions about their own and other’s emotions. This assumption is much less generally observable, and therefore must rely on the related theoretical foundations that have been constructed to this point. It is one thing to assume that children express emotions and parents and peers respond to emotions, but assuming that one parent or child responds differently to or feels differently about emotion than another parent or child is really where this theory becomes most useful. Without assuming that these differences in perspective and response exist, this theory would be of little utility. However, there is enough theoretical evidence established to this point to make the existence of variations in emotion-related responses a reasonable assumption. To illustrate the existence of this assumption, a number of studies have shown that parents perceive emotion differently depending on their own background and upbringing [3,5]. Moreover, studies have shown that parents tend to value or disvalue certain emotional expressions or displays depending on a number of factors including parental modeling, culture, psychopathology, and the emotional climate of the family [44,53,58]. For example, in many western cultures, sadness is considered to be more of a feminine emotion and anger more masculine. However, these perceptions of these emotions are not constant. It has been reported in some cultures that anger expression can been considered valuable for females, as it represents strength and the ability to protect oneself [59]. An additional example illustrating this assumption can be observed in the literature suggesting that along with placing different levels of value on various emotions, parents also tend to respond differently to the emotions of their child depending on a number of factors, including the emotion itself, the parent’s perceived appropriateness of the emotion, the child’s temperament, and the developmental stage of the child [5,60]. For example, a parent may have greater difficulty tolerating their eight-year-old sobbing loudly over a missing toy, compared to their two-year-old displaying the same behavior in a similar situation. Referring back to Sameroff’s Transactional Model and Bronfenbrenner’s PPCT Model, it is clear that emotion socialization is a bidirectional phenomenon that depends on the characteristics of both respective parties in parent–child and peer-child interactions [15,16]. Finally, it is important to recognize the role that psychopathology plays in responsivity styles, as a wealth of literature has shown that parenting styles can be influenced by psychopathological risk, both present and not present [9,58,61].

## 5. Research Methods for Implementation

Researching the existence of this theoretical typology would best be carried out using a mixed methods approach. As a starting point to assess emotion socialization patterns, researchers could consider the use of parent, child, and peer self-report questionnaires such as the Emotions as a Child Scale [62] or the Coping with Children’s Negative Emotions Scale [63]. Validated scales such as these have been used to capture responses to emotions in both children and caregivers. For the purposes of evaluating PPERS, developing a new questionnaire with parent and peer versions would prove beneficial. One additional self-reporting method that could be useful is experience sampling, where researchers capture an individual’s report on their current state or experience at multiple points throughout a given period rather than collecting an aggregate that would typically be reported in a questionnaire [18]. While insufficient alone in this context, such self-report measures can be particularly useful as they tend to capture an individual’s subjective experience with a given emotion without being influenced by modulation [18]. In addition to self-report methods, researchers might consider the use of emotion-based open-ended interviews in an attempt to capture the feelings of the parent or child, their reactions to their feelings, and their explanation for why they may respond to certain emotions or emotional behavior in a particular way [6,64]. Observing and coding emotion-based social interactions between parents and peers could be an additional method for evaluating the existence of this typology. These social interactions could be discussion based, or a simulation an emotional scenario between parent and child or child and peer [44]. Studies that have employed observational coding systems enable researchers to assess facial expressions, vocal tone, and body language as indicators of emotional response [65,66]. Additionally, researchers might consider reviewing observational recordings with the participant immediately following a recorded interaction to capture additional context or background of a particular moment [67]. Moreover, parent–child and child–peer conversations about emotions would be an important source for capturing how children form their understanding and internal working models of emotional responsivity. Using these bottom-up approaches can also be helpful for capturing the subtleties of emotion socialization in different cultures and contexts [6]. Finally, one might consider the use of psychophysiological methods by capturing emotion-related responses as they occur. Studies using EEG, fMRI, and electrodermal and respiratory measurement have shown to be effective for evaluating emotion socialization practices in both parents and children [68,69,70,71].

In sum, there are numerous methods that could be used to capture and evaluate the presence of these emotion responsivity styles in parents, children, and peers. Similar to the general findings of the early attachment styles [72,73] where roughly 65% percent are considered “secure” with notable variation in the remaining styles, we would not expect to see an equal distribution of these emotion responsivity styles across the population. It would be reasonable to expect some to be much more prevalent than others, especially considering how some PPERS tend to share common characteristics with traditional parenting styles. For example, Emotion Constructive and Emotion Responsive parents would also likely be identified as being authoritative. We would expect, however, that each style would be present to an extent in a representative sample. 

## 6. Implications and Conclusions

There are a number of notable implications associated with this theory. As emotion socialization continues to be increasingly recognized as a key source for optimal development throughout childhood and across the lifespan, this theory could aid parents in understanding the significance of their role as their child’s first teachers and emotion models, as well as how their emotional responses influence the emotion responsivity of their own children and in turn, their peers. Children possess increased vulnerability to potentially damaging emotion responsivity styles that can shape their perception of emotions and guide their social and emotional development in ways that can be maladaptive, including areas of psychopathology [9,10,11,12]. If parents could become aware of their own emotion responsivity styles, educated regarding the responsivity styles that they were exposed to as children, and taught what they could be doing to better assist their child’s emotional development, parents would have the potential to play an active and nurturing role in the emotional socialization and development of their children, and the transmission of healthy emotion responsivity styles from parent to child and child to peers. This development of this theory also has implications for clinicians, as children receiving therapy for emotion regulation problems could benefit from this typology by both assessing and addressing the emotion socialization process of the child within their family system. For example, by identifying that a child has been reared in a household where parents employ an Emotion Punitive style, clinicians may be better informed regarding the social and emotional deficiencies the child has experienced, as well as where there may be greater risk for psychopathology and need for intervention. 

If this theoretical typology proves to be useful, we would hope to be able to spread knowledge through further research and validation, policy development that can establish reliable channels for the spread and implementation of this valuable information, and ultimately the development of practices or tools to help parents recognize how they might be able to apply this information into their parenting behaviors. If researchers and policy makers could begin establishing and promoting the important role that both parents and peers play in the child’s emotional development, and how they nurture or hamper their progress, there may be a possibility for progress and positive change in the lives of families. In this case, generating knowledge of these responsivity types and their potential consequences, could prove powerful. Moreover, future studies could begin looking at the nuances in parent- and peer-responsivity in contexts of culture, family composition, psychopathological risk (present vs. not present), comparisons between motherhood and fatherhood, etc. If nothing else, we hope that the presentation of this theoretical typology will promote further theory development and research activity around various emotion socialization pathways, especially considering how parents emotionally socialize their children and how their children socialize their peers.
